# Chronic constipation in people with intellectual disabilities in the community: cross-sectional study

**DOI:** 10.1192/bjo.2024.12

**Published:** 2024-03-01

**Authors:** Richard Laugharne, Indermeet Sawhney, Bhathika Perera, Delia Wainwright, Paul Bassett, Briony Caffrey, Maire O'Dwyer, Kirsten Lamb, Mike Wilcock, Ashok Roy, Katy Oak, Sharon Eustice, Nick Newton, James Sterritt, Ruth Bishop, Rohit Shankar

**Affiliations:** Cornwall Partnership NHS Foundation Trust, Truro, UK; and Cornwall Intellectual Disability Equitable Research (CIDER), University of Plymouth, Truro, UK; Hertfordshire Partnership University NHS Foundation Trust, Hatfield, UK; Department of Psychiatry, University College London, London, UK; Devon Partnership NHS Trust, Exeter, UK; Statsconsultancy, Amersham, UK; Cornwall Partnership NHS Foundation Trust, Truro, UK; School of Pharmacy and Pharmaceutical Sciences, Trinity College, Dublin, Republic of Ireland; Cornwall Intellectual Disability Equitable Research (CIDER), University of Plymouth, Truro, UK; Royal Cornwall Hospitals Trust, Truro, UK; Coventry and Warwickshire Partnership NHS Trust, Birmingham, UK

**Keywords:** Constipation, bowel problems, premature mortality, developmental disabilities, polypharmacy

## Abstract

**Background:**

One-third to half of people with intellectual disabilities suffer from chronic constipation (defined as two or fewer bowel movements weekly or taking regular laxatives three or more times weekly), a cause of significant morbidity and premature mortality. Research on risk factors associated with constipation is limited.

**Aims:**

To enumerate risk factors associated with constipation in this population.

**Method:**

A questionnaire was developed on possible risk factors for constipation. The questionnaire was sent to carers of people with intellectual disabilities on the case-loads of four specialist intellectual disability services in England. Data analysis focused on descriptively summarising responses and comparing those reported with and without constipation.

**Results:**

Of the 181 people with intellectual disabilities whose carers returned the questionnaire, 42% reported chronic constipation. Constipation was significantly associated with more severe intellectual disability, dysphagia, cerebral palsy, poor mobility, polypharmacy including antipsychotics and antiseizure medication, and the need for greater toileting support. There were no associations with age or gender.

**Conclusions:**

People with intellectual disabilities may be more vulnerable to chronic constipation if they are more severely intellectually disabled. The associations of constipation with dysphagia, cerebral palsy, poor mobility and the need for greater toileting support suggests people with intellectual disabilities with significant physical disabilities are more at risk. People with the above disabilities need closer monitoring of their bowel health. Reducing medication to the minimum necessary may reduce the risk of constipation and is a modifiable risk factor that it is important to monitor. By screening patients using the constipation questionnaire, individualised bowel care plans could be implemented.

People with intellectual disabilities die on average 20 years earlier than the general population.^1^ Although respiratory and cardiovascular conditions are the leading causes of death, many other conditions, such as constipation, contribute to this premature mortality.^2^

## Constipation in people with intellectual disabilities

Constipation is a heterogeneous condition with multiple aetiology.^3^ Most prevalence rates of constipation in people with intellectual disabilities vary between 25 and 50%.^4^ Of 31 studies in a recent systematic review, constipation rates of over 33% were reported in 21 studies.^5^ There is a general acceptance that rates are higher than in the general population, with a quarter of people with intellectual disabilities prescribed repeated laxatives in a 12-month period, compared with 0.1% of the general population.^6^

Despite constipation being a common issue, it continues to be a significant problem in people with intellectual disabilities, often causing suffering and even leading to death.^7,8^ Hospital admission rates and mortality rates are difficult to estimate because of the varying methodologies by which they are recorded.^4^ Therefore, the evidence base is primarily from case reports, which have been described in the UK, Germany and Serbia,^4^ and a case series of 12 people with intellectual disabilities who died of constipation between 2015 and 2018.^1^

## Factors associated with constipation in intellectual disability

People with intellectual disabilities have several risk factors for developing problems with constipation.^5^ Factors associated with constipation in people with intellectual disabilities from previous studies include profound intellectual disability, cerebral palsy, lack of mobility, Down syndrome, certain medications (including anti-epileptic medication, benzodiazepines, histamine H_2_-receptor antagonists and proton pump inhibitors) and non-ambulatory status.^9^

## Current challenges in assessment of constipation-related risk factors

The commonly used diagnostic checklists, including the Rome IV checklist and Bristol Stool Chart, are focused on identifying constipation and not the risk factors for constipation specific to people with intellectual disabilities. These checklists can be difficult to use because people with intellectual disabilities may not have the cognitive ability to use them directly. It can be challenging for carers to bring detailed insight and information to such a personal matter of toileting, especially for individuals who are faecally incontinent and those who are non-verbal, thus resulting in poor data acquisition. There is a need for prospective studies to further clarify risk and mitigating factors. Evidence-based frameworks are needed for staff to consult in order to produce individualised programmes of bowel management.^10^

We aimed to survey the carers of people with an intellectual disability to identify the presence or absence of constipation in those they cared for and identify the presence of possible risk factors for constipation.

## Method

We followed the Strengthening the Reporting of Observational Studies in Epidemiology (STROBE) guidance for cross-sectional studies (www.strobe-statement.org/).

### Survey tool

Constipation was defined as opening bowels two or fewer times per week *or* using laxatives three or more times weekly. This is a definition used in another study on people with intellectual disabilities, including severely disabled people who are non-verbal and/or incontinent of faeces.^9^

The questionnaire was devised from the literature and a consensus of a group of experts including psychiatrists working with people with intellectual disabilities, pharmacists, a dietician, a specialist bowel nurse and a general practitioner (Supplementary Appendix, available at https://dx.doi.org/10.1192/bjo.2024.12). The focus of the questionnaire was to detect the symptoms of constipation and the presence or absence of possible risk factors for constipation. The questionnaire was devised such that it could be completed on paper, over the telephone or digitally. The target audience for the questionnaire were families and non-clinical professional carers. The draft survey was shared with the Inclusive Communication team in Cornwall County Council, a team dedicated to promoting and supporting delivery of effective communication. They provided guidance on inclusive communication measures and further adaptations to the survey. It was made explicit that this questionnaire is for carers of people with intellectual disabilities and not for people with intellectual disabilities themselves.

### Population and sample

The questionnaire was sent to the carers of all patients on the case-loads of four community intellectual disability teams across southern England, in Cornwall (population 538 000), Devon (750 000), Haringey (300 000) and Essex (population 500 000). The survey was disseminated via email and/or post, along with a cover letter and, if postal, a stamped return envelope.

### Ethics and governance

The authors assert that all procedures contributing to this work comply with the ethical standards of the relevant national and institutional committees on human experimentation and with the Helsinki Declaration of 1975, as revised in 2008. The National Health Service (NHS) Health Research Authority tool (www.hra-decisiontools.org.uk/research/index.html) confirmed that no formal NHS ethics approval was required (Supplementary information 2). The project was registered as a quality improvement project at uk.lifeqisystem.com. Each participating site registered the project at their respective NHS trust as a service evaluation/improvement project. Only the authors who worked for the respective intellectual disabilities services had access to any patient-identifiable information. Participants were informed in the cover letter that return of their survey via the pre-paid return envelope constituted informed consent to use the information provided. Collected clinical data at each NHS site were stored on an Excel spreadsheet, anonymised and then shared for analysis. The project used anonymised pooled data from the four centres, with each site having conducted a Data Protection Impact Assessment (DPIA) with support from their governance departments.

### Analysis

The analysis of the data focused on summarising the carers’ responses for the total sample of people with intellectual disabilities (‘participants’), and also comparing results between participants with and without constipation. The anticholinergic burden score was calculated from medication listed as taken by the participant,^11^ summarised and compared. Questionnaire responses were categorical in nature. These factors were compared between those with constipation and those without constipation using the chi-squared test. Fisher's exact test was used to determine whether there are non-random associations between two categorical variables. Continuous measurements were found to have a skewed distribution and the Mann–Whitney test was used to compare between the two groups. Significance was taken at *P* < 0.05.

## Results

The questionnaire was sent to the carers of individuals on the case-loads of four intellectual disability teams (approximately 800 people) and we received responses for 181 of these individuals. Responses on the two questions relating to presence or absence of constipation and/or laxative use are summarised in Table 1. The figures are the number of responses in total, and then the number in each category.

**Table 1:** Summary of constipation variables from questionnaires regarding 181 participants with intellectual disabilities

Variable	Participants, *n*	Category	Proportion of sample, *n* (%)
Bowel movements	165	≤2 per week	22 (13%)
		>2 per week	143 (87%)
Laxatives	172	Never	95 (55%)
		<3 per week	19 (11%)
		3+ per week	58 (34%)
Constipation^a^	164	No	95 (58%)
		Yes	69 (42%)

a. Defined as ≤2 bowel movements per week *or* laxatives ≥3 times per week.

The results suggested that 13% of participants had two or fewer bowel movements per week. One-third (34%) had three or more laxative uses per week.

Overall, constipation was assessed in 164 of the 181 participant forms received. Constipation could not be assessed for 17 participants owing to missing values for one or both components of the definition. Of those where constipation could be assessed, the data suggested that 42% of all participants had constipation defined as two or fewer bowel movements per week or regular laxatives three or more times weekly.

Table 2 summarises the demographics of the participants, along with other comorbid health conditions. The figures reported are the number of responses in each group, along with the number and percentage of responses in each category. Comparisons between those with and without constipation was made.

**Table 2:** Demographics and health conditions

Variable	Category	All participants (*n* = 181)	No constipation (*n* = 95)	Constipation (*n* = 69)	
		*n* (%)	*n* (%)	*n* (%)	*P*^a^
Age, years	18–25	24 (13%)	11 (12%)	10 (15%)	0.12
	26–39	64 (36%)	40 (42%)	17 (25%)	
	40–60	66 (37%)	34 (36%)	27 (40%)	
	Over 60	25 (14%)	10 (11%)	13 (19%)	
Gender	Female	81 (45%)	38 (40%)	37 (54%)	0.07
	Male	98 (55%)	57 (60%)	31 (46%)	
Level of intellectual disability	Mild	36 (20%)	19 (20%)	10 (15%)	**0.002**
	Moderate	80 (45%)	51 (54%)	22 (32%)	
	Severe	62 (35%)	24 (26%)	36 (53%)	
Conditions	Down syndrome	13 (8%)	8 (9%)	3 (5%)	0.37
	Other genetic	12 (8%)	5 (6%)	7 (13%)	0.20
	Cerebral palsy	24 (15%)	7 (8%)	16 (27%)	**0**.**002**
	Epilepsy	65 (39%)	32 (36%)	30 (48%)	0.13
	Dysphagia	42 (26%)	13 (15%)	27 (43%)	**<0**.**001**
	Diabetes	16 (10%)	6 (7%)	9 (15%)	0.11
	Obesity	30 (19%)	18 (21%)	11 (19%)	0.74
	Psychotic	9 (6%)	4 (5%)	5 (10%)	0.24
	Anxiety/depression	61 (40%)	36 (42%)	20 (36%)	0.48
	Autism/ADHD	86 (54%)	52 (60%)	27 (47%)	0.14

ADHD, attention-deficit hyperactivity disorder.

a. Significant correlations based on *P* < 0.05 are indicated in bold.

There was no evidence of a difference in the age and gender of those with and without constipation. The constipation group had a higher proportion of females than the group without constipation, but the difference did not reach statistical significance.

There was a significant difference in the level of intellectual disabilities between those with constipation and those without constipation (*P* = 0.002). In the constipation group, over half (53%) had moderate to profound intellectual disabilities, compared with only a quarter (26%) in the group without constipation.

The majority of other health conditions did not vary significantly between groups. However, differences were observed for cerebral palsy (*P* = 0.002) and dysphagia (*P* < 0.001). Both of these conditions were more common in those with constipation compared with those without constipation. Cerebral palsy was observed in 27% of those with constipation, compared with 8% without. Dysphagia was present in 43% of patients with constipation, but in only 15% of those without constipation.

Information on the toilet habits and constipation-influencing behaviours is summarised in Table 3. The fluid intake of the two groups did not significantly differ. However, differences between groups were observed for toileting (*P* < 0.001), toilet routine (*P* < 0.001) and mobility (*P* < 0.001). Those with constipation were more likely to require toileting support, more likely to have an assisted toilet routine and had worse mobility compared with those without constipation. There was also evidence that those with constipation were more likely to use a footstool or other toileting aids, although this difference did not quite reach statistical significance (*P* = 0.07).

**Table 3:** Constipation-influencing behaviours

Variable	Category	All participants (*n* = 181)	No constipation (*n* = 95)	Constipation (*n* = 69)	
		*n* (%)	*n* (%)	*n* (%)	*P*
Fluid intake	Difficult	26 (15%)	16 (18%)	7 (10%)	0.20
	Good	144 (85%)	74 (82%)	60 (90%)	
Toileting	Independent	75 (57%)	55 (71%)	9 (24%)	**<0.001**
	Support required	56 (43%)	23 (29%)	28 (76%)	
Toilet seat	Normal seat	127 (88%)	77 (91%)	37 (82%)	0.17
	Raised seat	17 (12%)	8 (9%)	8 (18%)	
Use of footstool/aids	No	131 (91%)	80 (94%)	38 (84%)	0.07
	Yes	13 (9%)	5 (6%)	7 (16%)	
Toilet routine	No routine	104 (63%)	70 (78%)	27 (42%)	**<0**.**001**
	Assisted routine	61 (37%)	20 (22%)	37 (58%)	
Mobility	Good	117 (66%)	76 (81%)	28 (41%)	**<0**.**001**
	Impaired	15 (8%)	9 (10%)	4 (6%)	
	Largely immobile	35 (20%)	6 (6%)	29 (42%)	
	Not out of bed	10 (6%)	3 (3%)	7 (10%)	

a. Significant correlations based on *P* < 0.05 are indicated in bold.

Three-quarters (76%) of those with constipation required support with toileting, compared with only 29% of those without constipation. Less than half (41%) of those with constipation were reported to have good mobility, contrasting with more than three-quarters (81%) of those without constipation. These findings may be explained by greater physical disability among those with constipation.

Information on medications is summarised in Table 4. Overall, 41% of participants were on anti-epileptic medication, and one-third (33%) were on antipsychotics. These medications, along with benzodiazepines, H_2_ antagonists and proton pump inhibitors, did not vary significantly between those with and without constipation. However, the total number of medications was significantly higher in the constipation group. The group with constipation had a median of five medications, compared with a median of three for the group without constipation. The anticholinergic burden (ACB) scores of the two groups were not significantly different, although a higher score was found in those with constipation (ACB = 2) than those without (ACB = 1).

**Table 4:** Medications

Variable	Category	All participants (*n* = 181)	No constipation (*n* = 95)	Constipation (*n* = 69)	
		*n* (%)	*n* (%)	*n* (%)	*P*
Medication	Antipsychotics	60 (34%)	34 (37%)	19 (28%)	0.21
	Anti-epileptics	72 (41%)	36 (39%)	33 (48%)	0.27
	Benzodiazepines	33 (19%)	16 (17%)	15 (21%)	0.49
	H_2_ antagonists	0 (0%)	0 (0%)	0 (0%)	–
	Proton pump inhibitors	38 (22%)	21 (23%)	15 (22%)	0.87
Laxatives	Stool softener	0 (0%)	0 (0%)	0 (0%)	–
	Bulk forming	1 (1%)	0 (0%)	1 (1%)	0.43
	Osmotic	48 (27%)	5 (5%)	42 (61%)	**<0**.**001**
	Stimulant	17 (10%)	1 (1%)	16 (23%)	**<0**.**001**
	Any	51 (29%)	5 (5%)	45 (65%)	**<0**.**001**
Total medications^b^	–	4 (2, 6)	3 (1, 5)	5 (3, 7)	**<0**.**001**
ACB score^b^	–	2 (0, 3)	1 (0, 3)	2 (0, 4)	0.31

ACB, anticholinergic burden.

a. Significant correlations based on *P* < 0.05 are indicated in bold.

b. Summary statistics are: median (interquartile range).

Laxatives were significantly more common in those with constipation. This result is not surprising as laxative use was part of the definition of constipation. Over 65% of the constipation group used laxatives, compared with 5% of those without constipation. Osmotic laxatives were the most used in both groups. Both osmotic and stimulant laxatives had higher levels of use in the group with constipation than in those without constipation (*P* < 0.001).

## Discussion

Previous studies have suggested that the prevalence of constipation in people with intellectual disabilities is between 33 and 50%^5^ and the prevalence in this sample was 42%. The main findings of this community survey of people with intellectual disabilities is that those who were constipated were more likely to have greater severity of both physical disability as well as intellectual disability. They were more likely to have cerebral palsy, experience dysphagia, have poor mobility and require an assisted toileting regime. They were also more likely to be on a greater number of medications. The association with severity of the intellectual disability and poor physical mobility is consistent with previous research^5,9^ but adds to it in emphasising the importance of dysphagia, severity of physical disabilities and polypharmacy as risk factors. The consistency in findings is, however, concerning as one of the main previous studies (in The Netherlands) was conducted over 20 years ago^9^ and the situation has changed little. Our study group devised a constipation questionnaire which has been shown to be feasible in routine clinical services and completed by carers with varying degrees of knowledge.

A concern is the high prevalence of anti-seizure medication and antipsychotics identified in our sample. Recent studies focused on characteristics of people with intellectual disabilities and epilepsy have highlighted the high frequency of antipsychotic prescribing along with associated polypharmacy.^12^ Furthermore, in a recent case–control study comparing a group of people with epilepsy and intellectual disability who had died with live controls from the same population, the authors identified significant differences in antipsychotic prescribing and overall polypharmacy as major modifiable factors.^13^ The links of these factors (antipsychotics, anti-epileptics and polypharmacy) to premature mortality in people with intellectual disabilities and their links in our study to constipation needs to be highlighted.

### Limitations

Our questionnaire was devised by experts in a wide range of fields and made use of the published literature. However, although the questionnaire has face validity it has not been tested for other psychometric properties. We cannot claim our sample to be representative of the intellectual disability population as the responses depended on the willingness of carers to complete the questionnaire. Further, the questionnaires are only in English and we failed to capture respondent and participant ethnicity, which could influence the feedback. Our study sample comprised individuals under the care of secondary specialist community services for people with intellectual disabilities and therefore will not reflect people with intellectual disabilities not under the care of secondary services and solely cared for in primary care. It could be considered a convenience sample. The study population was selected through clinical networks and is not necessarily representative of the whole population. However, it includes urban and rural communities in the south of England.

We included all questionnaires that were sent back and could not monitor response rates or the characteristics of those who did and did not respond. Therefore, we cannot claim that those responding are representative of all patients on the case-loads of the secondary specialist teams.

### Implications for clinical practice

Our study suggests that people with more severe intellectual disabilities, more severe physical disabilities (including dysphagia and cerebral palsy) and on multiple medications are more likely to suffer from constipation. This subset of people with intellectual disabilities need vigilance over their bowel health. Regular and possibly enhanced screening, together with an individualised bowel health plan, may benefit them. This should be coupled with regular medication reviews, especially if there is polypharmacy and particularly if the polypharmacy is suspected to affect bowel health. The Annual Health Checks could be an important vehicle to provide a basic and essential level of oversight on these matters for this vulnerable cohort.

It is recognised that carers and family members value good person-centred communication from professionals on constipation management.^14^ The risk factors identified could provide improved feedback from clinicians to carers.

The finding that 181 carers were able to complete the questionnaire and the findings were coherent suggests that it is feasible to use this questionnaire in clinical practice to detect constipation and identify risk factors that can be modified, although further research needs to be conducted to establish whether it can lead to changes in outcomes. Individualised bowel care plans may be based on the feedback from the questionnaire, and this has been delivered for some patients.

### Implications for research

Although there has been significant work done in this area and reviews of the literature, the focus has been more on understanding the broader problem than specific influencers. We suggest the following:

- there is a need for large-scale studies using multivariate analysis to further clarify the modifiable and non-modifiable risk factors associated with constipation in people with intellectual disabilities;
- constipation questionnaires and individualised bowel care plans need to be evaluated to examine whether they improve bowel health and quality of life, which should be the goal of our endeavours;
- targeted screening and interventions for people with intellectual disabilities most vulnerable to constipation, particularly those with severe intellectual and physical disabilities, should be tested and interventions examined;
- implementation science needs to be utilised to enable research findings to be implemented in routine clinical care and not just in research arenas.

### Implications for policy

Clear guidelines are needed on monitoring, detecting and treating constipation in people with intellectual disabilities, emphasising the greater vulnerability of those with severe physical disabilities.

## Supporting information

Laugharne et al. supplementary material Laugharne et al. supplementary material

## Data Availability

The data that support the findings of this study are available from the corresponding author on reasonable request.
